# Biosynthesis of Glycine from One-Carbon Resources Using an Engineered *Escherichia coli* Whole-Cell Catalyst

**DOI:** 10.3390/microorganisms14010236

**Published:** 2026-01-20

**Authors:** Muran Fu, Hongling Shi, Xueyang Bai, Qian Gao, Fei Liu, Dandan Li, Yunchao Kan, Chuang Xue, Lunguang Yao, Cunduo Tang

**Affiliations:** 1 Henan Provincial Engineering Laboratory of Insect Bio-Reactor, Henan International Joint Laboratory of Insect Biology and Henan Key Laboratory of Insect Biology, College of Life Science, Nanyang Normal University, 1638 Wolong Road, Nanyang 473061, China; 2023086001003@nynu.edu.cn (M.F.); 20142041@nynu.edu.cn (H.S.); 2024086001003@nynu.edu.cn (X.B.); 22540140217@nynu.edu.com (Q.G.); 20081059@nynu.edu.cn (F.L.); 20112048@nynu.edu.cn (D.L.); 20131007@nynu.edu.cn (Y.K.); 2 School of Bioengineering, Dalian University of Technology, 2 Linggong Road, Dalian 116024, China; xue.1@dlut.edu.cn

**Keywords:** carbon dioxide reduction, whole-cell catalysis, one-pot synthesis, whole-cell electrocatalysis

## Abstract

Carbon dioxide (CO_2_) is a cost-effective, abundant, and renewable carbon source, but its utilization technologies face several issues. The reductive glycine pathway (RGP) is recognized as one of the most efficient one-carbon (C1) assimilation routes in nature, with its core component—the glycine cleavage system (GCS: GcvP, GcvH, GcvT, and GcvL)—playing an essential role in C1 metabolism. To develop efficient CO_2_ conversion and utilization pathways, we identified *Nh*FtfL and *Am*FchA-MtdA with high catalytic efficiency through gene mining and constructed a four-plasmid co-expression system in *E. coli* BL21(DE3) using Gibson Assembly. This system integrated GcvP-GcvH, GcvT-GcvL, *Nh*FtfL-*Am*FchA-MtdA, and *Rs*PPK2, thereby reconstituting the complete RGP while enhancing ATP supply. The engineered strain functioned as an efficient whole-cell biocatalyst, achieving a glycine space–time productivity of 0.125 mmol/L/h via one-pot conversion of formate. Furthermore, we expanded the application scope by developing a whole-cell electrocatalysis system that directly synthesized glycine from CO_2_ and NH_4_Cl, achieving a glycine space–time productivity of 0.135 mmol/L/h. This study demonstrates the potential of the engineered RGP system for upgrading C1 resources and supports the transition toward carbon neutrality.

## 1. Introduction

The rapid industrialization and significant increase in urban emissions in China in recent years have exacerbated climate degradation and environmental pollution, posing a serious threat to human survival [1,2]. However, carbon dioxide (CO_2_) is a cost-effective, abundant, and renewable carbon source that can be utilized for producing fuels and basic chemicals such as formic acid, methanol, and glycine [3]. Consequently, converting CO_2_ into fuels and chemicals represents a cutting-edge strategy for achieving a carbon cycle, combating global warming, and alleviating the energy crisis [4]. The conversion of CO_2_ involves thermodynamic and kinetic challenges [5]. Current CO_2_ utilization technologies face several issues: short sequestration periods, limited emission reduction, weak techno-economic viability, and additional energy consumption [6]. Therefore, achieving more efficient CO_2_ conversion requires long-term research [7]. Electrocatalysis often suffers from low selectivity, a wide range of by-products, low current density, and reduced efficiency [8]. Biocatalysis, which utilizes living cells or enzymes as catalysts to synthesize complex organic molecules from CO_2_, offers high selectivity, environmental friendliness, and mild operating conditions, and it facilitates carbon–carbon coupling and chain extension, compared to chemical catalysts [9]. However, the high chemical inertness of CO_2_ and difficulties in regulating product selectivity present significant challenges, as breaking the C=O bond requires substantial energy input, complicating activation and bond cleavage. To address this, enzymatic electrocatalysis of CO_2_ has been proposed.

Enzymes utilized for CO_2_ conversion into chemicals encompass oxidoreductases, such as formate dehydrogenase (FDH), carbon monoxide dehydrogenase (CODH), and CO_2_ reductases, as well as pyruvate decarboxylase and carbonic anhydrase [10]. Although the application of pyruvate dehydrogenase and carbonic anhydrase is often limited by high costs or challenges in achieving desired outcomes, FDH can be integrated into multi-enzyme cascade systems—for instance, cooperating with formaldehyde dehydrogenase (FaldDH) and alcohol dehydrogenase (ADH) to generate higher-value chemicals and fuels. Successful examples to date include metal or NADH-dependent FDHs reducing CO_2_ to formate [11]; nickel-dependent CODHs producing CO [12]; and VFe nitrogenases synthesizing methane, ethylene, and propane [13]. More remarkably, a series of enzymes can cooperate to form complex biochemical pathways, thereby extending carbon chain length and upgrading fixed CO_2_. For instance, a cycle converting CO_2_ to oxalate achieves temporary carbon chain elongation of intermediates through sequential carboxylation reactions [14]. Schwander et al. [13] demonstrated continuous in vitro CO_2_ fixation into organic molecules (e.g., glyoxylate), extending the carbon chain from a three-carbon (propionyl-CoA) to a four-carbon unit. Meanwhile, Cai et al. [15] constructed an artificial starch synthesis pathway (ASAP) that achieves carbon chain extension via multi-step enzymatic reactions: starting from methanol as a C1 precursor and progressively elongating from C1 to Cn. Notably, this process requires chemical hydrogenation to reduce CO_2_ to methanol, resulting in a stepwise rather than one-pot reaction system. Recently, the electrocatalytic synthesis of C-N-coupled compounds from greenhouse gas CO_2_ and abundant nitrogen-containing substances has attracted significant scientific attention [16]. These emerging approaches have successfully produced higher-value C-N-coupled products such as urea and amines, broadening the scope of accessible products. However, research on amino acid synthesis remains relatively limited.

**Figure 1:**
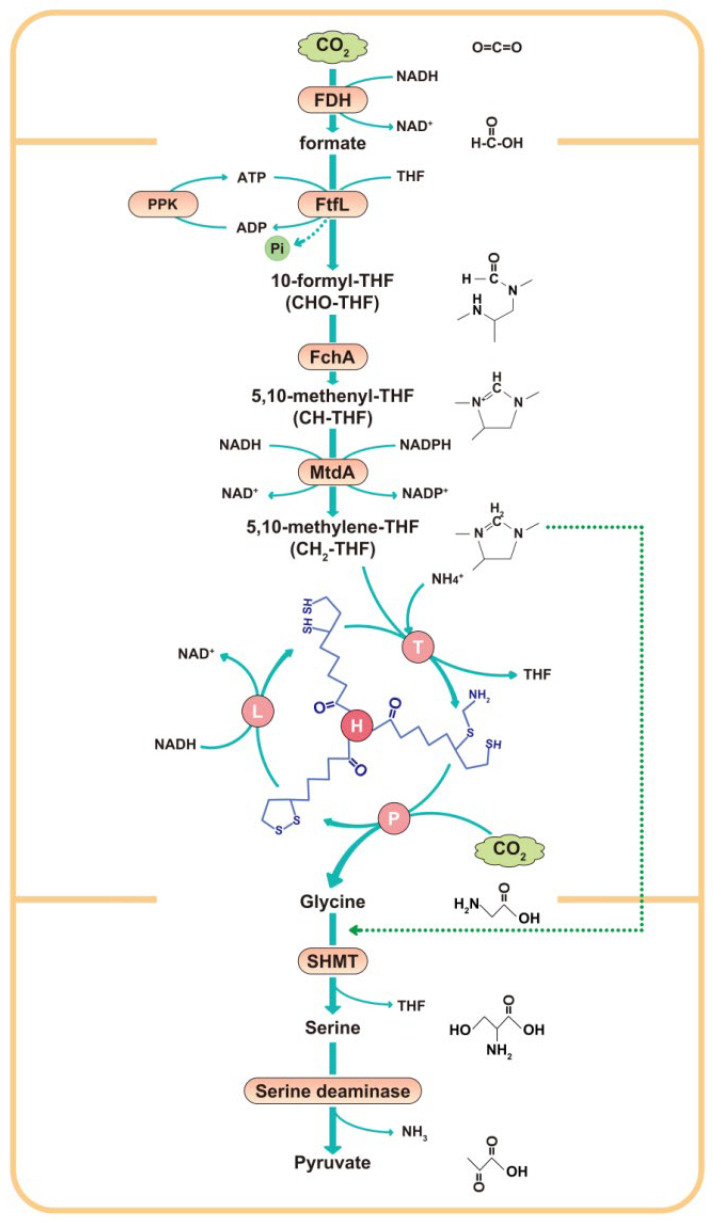
**The reductive glycine pathway.** FDH, formate dehydrogenase; FtfL, formate–tetrahydrofolate ligase; FchA, methenyltetrahydrofolate cyclohydrolase; MtdA, methylenetetrahydrofolate dehydrogenase; P, H, T, and L, glycine cleavage system (GCS: GcvP, GcvH, GcvT, and GcvL); PPK, polyphosphate kinase; THF, tetrahydrofolate; SHMT, serine hydroxymethyltransferase. This study focused on glycine synthesis from formate, with individual validation of both the glycine cleavage system (GCS: GcvP-GcvH-GcvT-GcvL) and the reductive glycine pathway (RGP: *Nh*FtfL-*Am*FchA-MtdA-GcvP-GcvH-GcvT-GcvL).

The catalytic system based on FDH offers a novel strategy for valorizing C1 resources, leveraging its distinctive advantages, including mild reaction conditions, low operational costs, and capability to enable carbon cycling coupled with energy conversion. The biological utilization pathways of formate can be categorized into upstream assimilation (conversion of formate to 5,10-methylenetetrahydrofolate (5,10-methylene-THF) and downstream assimilation (conversion of 5,10-methylene-THF to metabolically utilizable compounds). Downstream metabolic pathways are primarily divided into three types: the Serine Cycle, the Wood–Ljungdahl Pathway, and the RGP. Kim et al. [17] successfully constructed an artificial pathway in *Escherichia coli* (*E. coli*) that converts formate to phosphoglycerate via the Serine Cycle. By integrating this pathway with adaptive laboratory evolution, they achieved the production of ethanol from formate. However, the Serine Cycle exhibits significant overlap with central metabolism, complicating its rational design and engineering. Furthermore, this pathway is associated with high consumption of reducing equivalents and ATP, rendering it suboptimal for efficient formate utilization. Although the Wood–Ljungdahl Pathway operates with low energy consumption, it requires carbon monoxide (CO) for the synthesis of acetyl-CoA. The low solubility of CO in aqueous solutions results in relatively low formate assimilation efficiency in the Wood–Ljungdahl Pathway [18]. When formate is directed through the RGP, it undergoes stepwise metabolism to synthesize glycine, serine, and pyruvate. This pathway offers distinct advantages: most of its constituent enzymes are widely present across biological systems, it exhibits minimal overlap with central metabolism, and it has low energy demands. Consequently, the RGP is recognized as the most efficient pathway for high-yield formate utilization. Specifically (Figure 1), CO_2_ is reduced to formate under the catalysis of FDH; then, formate is sequentially converted to 5,10-methylene-THF through the catalysis of formate–tetrahydrofolate ligase (FtfL), methylenetetrahydrofolate cyclohydrolase (FchA), and methylenetetrahydrofolate dehydrogenase (MtdA); and, finally, 5,10-methylene-THF is subjected to the glycine cleavage system (GCS), which consists of four protein components (GcvT, GcvH, GcvP, and GcvL). The GCS condenses 5,10-methylene-THF with another CO_2_ and NH_3_ molecule to produce glycine, which can also be further converted to serine via serine hydroxymethyltransferase (SHMT). Incorporating polyphosphate kinase 2 (PPK2) into this pathway serves dual essential functions: it supplies ATP for formate activation (formate → formyl-THF) while simultaneously maintaining the energy demands for reverse operation of the GCS [19].

CO_2_ + NH_3_ + 5,10-CH_2_-THF + NADH + H^+^ ⟷ Glycine + NAD^+^ + THF

In the enzymatic reaction cycle of GCS, the H protein (GcvH) is a protein containing a sulfur coenzyme. During the process of shuttling among the active centers of the other three catalytic proteins, the H protein forms three states with its covalent ligands, namely the oxidized state (H_ox_), the transitional state (H_int_), and the reduced state (H_red_) [20], and interacts with the other three proteins through its freely swinging lipoyl arm [21,22]. The P protein (GcvP) is a protein containing metal ions, which can catalyze the fixation and conversion of CO_2_ into amino acids. The T protein (GcvT) and L protein (GcvL) are auxiliary proteins that can interact with the H protein and P protein to promote electron transfer and the progress of the reaction [23]. These components work together to achieve the reaction that converts inorganic CO_2_ captured from the air and inorganic ammonia into organic glycine. This pathway not only enables CO_2_ fixation but also efficiently converts C1 units (e.g., CO_2_ and formate) into organic carbon skeletons, thereby facilitating the synthesis of diverse high-value products. Researchers have developed an electrochemical–biological hybrid system in which CO_2_ is first reduced to formate via electrocatalysis [24]. The formate, along with CO_2_, is subsequently assimilated by an engineered *E. coli* strain equipped with the RGP, supporting pyruvate synthesis and microbial growth. This system demonstrates the potential for producing chemicals from CO_2_ and electrical energy. Furthermore, Wu et al. [25] constructed an in vitro multi-enzyme cascade based on the RGP, achieving enzymatic electrocatalytic synthesis of glycine using CO_2_ and NH_3_ as the sole carbon and nitrogen sources. However, such in vitro free-enzyme systems face challenges related to poor environmental stability and limited reusability. In contrast, one-pot, whole-cell biocatalysis offers significant advantages: enzymes reside in their native cellular environment, protected by cellular membranes, cytoskeletal structures, and chaperone proteins, thereby maintaining structural integrity and activity over extended periods. Additionally, the spatial organization of multiple enzymes and cofactors within the cell enables direct channeling of intermediates, minimizing diffusion losses and enhancing overall reaction efficiency and selectivity.

This study initiated with gene mining to identify *Nh*FtfL and *Am*FchA-MtdA, which exhibit superior catalytic efficiency. Subsequently, a co-expression system comprising GcvP-GcvH, GcvT-GcvL, and *Nh*FtfL-*Am*FchA-MtdA was constructed in *E. coli* BL21(DE3). Through synergistic integration with *Rs*PPK2, we established an engineered whole-cell biocatalyst harboring four plasmids for the RGP. Furthermore, we demonstrated the feasibility of a one-pot, whole-cell catalytic synthesis of glycine from formate using this quadruple-plasmid recombinant *E. coli* strain, achieving a glycine titer of up to 0.75 mM. Expanding on this, we implemented a dual-strain-coupling strategy for the electrocatalytic synthesis of glycine directly from CO_2_ and NH_4_Cl. This system employed two engineered strains: a D533S/E684I [26] mutant *Pb*FDH-expressing *E. coli* for CO_2_ reduction to formate, and the quadruple-plasmid RGP strain for glycine synthesis. This research accomplishes the reconstruction of a highly complex metabolic pathway, establishing not only a streamlined one-pot in vivo process for converting formate to glycine but also a pioneering whole-cell electrocatalysis system for direct glycine production from CO_2_ and NH_4_Cl. Our work provides a significant reference for expanding CO_2_ utilization routes and contributes to advancing the carbon-neutrality goal.

## 2. Materials and Methods

### 2.1. Strains and Materials

The details of the strains and plasmids used in this study are provided in Table S1. The designations of some recombinant strains are listed in Table 1. Strain A (*E. coli* BL21/pACYCDuet-1-GcvP-GcvH: pETDuet-1-GcvT-GcvL), strain B (*E. coli* BL21/pCDFDuet-1-*Am*FchA-MtdA: pACYCDuet-1-GcvP-GcvH: pETDuet-1-GcvT-GcvL), strain C (*E. coli* BL21/pCDFDuet-1-*Nf*FchA-MtdA: pACYCDuet-1-GcvP-GcvH: pETDuet-1-GcvT-GcvL), strain D (*E. coli* BL21/pCDFDuet-1-*Nh*FtfL-*Am*FchA-MtdA: pACYCDuet-1-GcvP-GcvH: pETDuet-1-GcvT-GcvL), and strain E (*E. coli* BL21/RSFDuet-1-*Rs*PPK2: pCDFDuet-1-*Nh*FtfL-*Am*FchA-MtdA: pACYCDuet-1-GcvP-GcvH: pETDuet-1-GcvT-GcvL) were employed in the GCS or RGP to produce glycine in this study. The primers used are listed in Table S2.

### 2.2. Gene Cloning and Expression of Individual Enzyme in RGP

We obtained the gene sequences of the GCS from the NCBI database, as shown in Table 2 (GcvP: NCBI No. WP_112929453.1; GcvH: NCBI No. WP_001295377.1; GcvT: NCBI No. WP_099356926.1; GcvL: NCBI No. WP_110826218.1). Using the *E. coli* genome as a template, we successfully amplified GcvP, GcvH, GcvT, and GcvL through seamless cloning technology. Subsequently, GcvP and GcvH were each ligated to the pACYCDuet-1 vector, while GcvT and GcvL were each ligated to the pETDuet-1 vector. By using the engineered strain *E. coli* BL21(DE3), we achieved the heterologous expression of the GCS, obtaining recombinant strains *E. coli* BL21/pACYCDuet-1-GcvP, *E. coli* BL21/pACYCDuet-1-GcvH, *E. coli* BL21/pETDuet-1-GcvT, and *E. coli* BL21/pETDuet-1-GcvL. Subsequently, to optimize the multi-step reaction, GcvH was ligated to the multiple cloning site 2 (MCS2) site of the pACYCDuet-1-GcvP vector, and GcvL was ligated to the MCS2 site of the pETDuet-1-GcvT vector. These constructs were then transformed into *E. coli* BL21(DE3) competent cells, resulting in double-gene-expressing recombinant strains *E. coli* BL21/pACYCDuet-1-GcvP-GcvH and *E. coli* BL21/pETDuet-1-GcvT-GcvL. Finally, the plasmids pACYCDuet-1-GcvP-GcvH and pETDuet-1-GcvT-GcvL were introduced into the same *E. coli* BL21(DE3) competent cells, obtaining the recombinant *E. coli* strain known as strain A.

Subsequently, the synthesis of glycine using formate as a substrate was investigated. According to previous reports, the bifunctional enzyme *C. formicaceticum* FchA-MtdA exhibits higher glycine synthesis activity than the monofunctional enzymes FchA and MtdA [25]. Therefore, we identified five novel enzymes with varying degrees of similarity to this enzyme. Through multiple sequence alignment, two new enzymes—*Alkaliphilus metalliredigens* FchA-MtdA (*Am*FchA-MtdA: WP_012063162.1), with 90% similarity, and *Natronincola ferrireducens* FchA-MtdA (*Nf*FchA-MtdA: WP_090553537.1), with 88% similarity—were selected for experimentation. The multiple sequence alignment of FchA-MtdA is shown in Figure S1. Similarly, FtfL from *Neomoorella humiferrea* (*Nh*FtfL: WP_338835031.1) was also identified and selected. Using genetic engineering techniques, *Am*FchA-MtdA and *Nf*FchA-MtdA were separately digested and ligated into the MCS2 site of the pCDFDuet-1 vector, which was then transformed into *E. coli* to obtain the recombinant strains *E. coli* BL21/pCDFDuet-1-*Am*FchA-MtdA and *E. coli* BL21/pCDFDuet-1-*Nf*FchA-MtdA. Subsequently, *Nh*FtfL was digested and ligated into the MCS1 site of the pCDFDuet-1-*Am*FchA-MtdA vector and transformed into *E. coli*, yielding the recombinant strain *E. coli* BL21/pCDFDuet-1-*Nh*FtfL-*Am*FchA-MtdA. Strain A was prepared as competent cells. The plasmids pCDFDuet-1-*Am*FchA-MtdA, pCDFDuet-1-*Nf*FchA-MtdA, and pCDFDuet-1-*Nh*FtfL-*Am*FchA-MtdA were then introduced into these competent cells via heat shock, resulting in the recombinant *E. coli* strains strain B, strain C, and strain D. Finally, PPK2 from *Rhodobacter sphaeroides* (*Rs*PPK2: UniProt No. Q3IZT9) was digested and ligated into the RSFDuet-1 vector, which was then transformed into *E. coli* to obtain the recombinant strain *E. coli* BL21/RSFDuet-1-*Rs*PPK2. Strain D was prepared as competent cells, and RSFDuet-1-*Rs*PPK2 was introduced via heat shock, yielding the recombinant *E. coli* strain known as strain E (Figure S2).

The recombinant cells were cultured in LB medium supplemented with 0.1% corresponding antibiotics (50 μg/mL kanamycin, 100 μg/mL ampicillin, 25 μg/mL chloramphenicol, and 50 μg/mL streptomycin) and 2% inoculum at 37 °C with 200 rpm shaking until *OD*_600_ reached 0.6–0.8. Protein expression was then induced by adding 0.1 mM IPTG, followed by incubation at 16 °C with 200 rpm shaking for 20 h. The induced cells were lysed using an ultrasonic disruptor, followed by centrifugation at 4 °C and 12,000 rpm for 15 min to collect the crude protein. The protein was purified using affinity chromatography (Ni-Agarose resin), and the protein concentration was determined by the Bradford method. Additionally, sodium dodecyl sulfate–polyacrylamide gel electrophoresis (SDS-PAGE) was performed using a 12.5% gel, and the apparent molecular weight of the protein was calculated using Quantity One V4.6.6 software.

### 2.3. Enzyme Activity Assay and Enzymatic Characterization

The redox activities of GCS and GcvP were measured by monitoring the concentration of NADH during the reaction. Prior to the reaction, the following reaction mixture (excluding the enzyme solution) was stored at a mild temperature for 3 min. The oxidative and reductive activities were determined by monitoring the formation or depletion of NADH at 340 nm for 1 min using a microplate reader or UV spectrophotometer. Under these conditions, the amount of enzyme required to form or consume 1 μmol NADH per minute (ε_NADH,340 nm_ = 6220 mol^−1^·cm^−1^) was defined as one unit of oxidative or reductive activity. Enzyme activity was expressed as follows:

U = EW·V·1000/6220·L = EW/6.22

where EW represents the absorbance change per minute. In this study, the molar extinction coefficient was set at 6220 L·mol^−1^·cm^−1^, with V and L denoting the reaction volume (mL) and path length (cm), respectively.

The activity of GCS in catalyzing glycine cleavage was measured at 37 °C by monitoring glycine-dependent NADH formation at 340 nm using a microplate reader. The reaction system contained Tris-HCl (50 mM, pH 7.5), 0.5 M THF, 20 mM DTT, 25 μM PLP, 5 mM NAD^+^, 5 μM GcvP, 5 μM GcvT, 5 μM GcvL, and 10 μM GcvH (or 10 μM GcvP-GcvH-GcvT-GcvL). The mixture was centrifuged at 12, 000 rpm for 1 min. Then, 190 μL of the premix was added to a 96-well plate, and the reaction was initiated by adding 10 μL of glycine (final concentration: 50 mM) [21].

To measure the activity of GcvP in catalyzing glycine cleavage, GcvP was made the rate-limiting step of the whole system and combined with saturating amounts of GcvT, GcvH, and GcvL. The enzymatic activity of P protein was determined by measuring the formation of NADH at 340 nm, at 37 °C, using a microplate reader. The reaction mixture included Tris-HCl (50 mM, pH 7.5), 0.5 mM THF, 20 mM DTT, 25 μM PLP, 5 mM NAD^+^, 0.01 μM GcvP, 5 μM GcvT, 5 μM GcvL, and 10 μM GcvH. After premixing, the reaction was initiated by adding glycine (final concentration: 50 mM).

The activity of GCS in catalyzing glycine synthesis was assayed at 30 °C, and the reduction activity was determined by monitoring the decrease in NADH at 340 nm for 1 min using a UV spectrophotometer. The reaction system contained 100 mM sodium phosphate buffer (pH 7.0), 10 mM formate, 50 mM NaHCO_3_, 50 mM NH_4_Cl, 2 mM THF, 2 mM NADH, 3 mM lipoic acid, 5 mM ATP, 0.25 mM PLP, 10 mM DTT, 5 mM MgCl_2_, and 1 mg·mL^−1^ of *Nh*FtfL-*Am*FchA-MtdA. After premixing, the reaction was initiated by adding 0.01 μM GcvP-GcvH-GcvT-GcvL.

The activity of GcvP in catalyzing glycine synthesis was assayed at 30 °C, and the reduction activity was determined by monitoring the decrease in NADH at 340 nm for 1 min using a UV spectrophotometer. The reaction system contained 100 mM sodium phosphate buffer (pH 7.0), 10 mM formate, 50 mM NaHCO_3_, 50 mM NH_4_Cl, 2 mM THF, 2 mM NADH, 3 mM lipoic acid, 5 mM ATP, 0.25 mM PLP, 10 mM DTT, 5 mM MgCl_2_, 1 mg·mL^−1^ of *Nh*FtfL-*Am*FchA-MtdA, 5 μM GcvT, 5 μM GcvL, and 10 μM GcvH. After premixing, the reaction was initiated by adding 0.01 μM GcvP.

To determine the optimal temperature for GcvP-catalyzed glycine production, we set a temperature range from 30 to 70 °C (at 5 °C intervals) and conducted activity assays under standard conditions. We considered the residual enzyme activity at the optimal temperature to be 100% to investigate the optimal temperature of free GcvP.

To evaluate the stability of free GcvP at different temperatures, free GcvP was incubated at 20–55 °C (at 5 °C intervals) for 1 h. Then, the residual enzyme activity was measured at the optimal reaction temperature. The residual enzyme activity after storage in an ice bath for 1 h was taken to be 100% to investigate the temperature stability of free GcvP.

### 2.4. Synthesis of Glycine by GCS

To validate the GCS, we involved the free enzymes of the GCS (GcvP, GcvH, GcvT, GcvL) and strain A in the reaction, respectively. Xu et al. [21] investigated the kinetic behavior of GCS and demonstrated the presence of a large amount of formaldehyde during the GCS reaction. To reduce the impact of the byproduct formaldehyde on the enzymes, DTT (dithiothreitol) was added to the reaction system. The reducing agent DTT can not only enhance the tolerance of GCS to formaldehyde but also act as an activator to increase the synthesis rate of glycine and promote the reaction [27]. The reaction mixture (1 mL) contained 100 mM sodium phosphate buffer (pH 7.0), 1 mM 5,10-methylene-THF, 50 mM NaHCO_3_, 50 mM NH_4_Cl, 1 mM NADH, 3 mM lipoic acid, 0.25 mM PLP, 10 mM DTT, 5 mM MgCl_2_, 200 μg·mL^−1^ GcvT, 200 μg·mL^−1^ GcvH, 400 μg·mL^−1^ GcvP, and 200 μg·mL^−1^ GcvL (or 400 μg·mL^−1^ GcvP-GcvH-GcvT-GcvL). The reaction temperature was maintained at 30 °C.

### 2.5. Synthesis of Glycine Using Strain A

To verify the RGP, we used strain A (GcvP-GcvH-GcvT-GcvL), along with the free enzymes *Am*FchA-MtdA and *Nf*FchA-MtdA, to catalyze the formation of glycine from formate. The reaction mixture (1 mL) contained 100 mM sodium phosphate buffer (pH 7.0), 10 mM formate, 50 mM NaHCO_3_, 50 mM NH_4_Cl, 2 mM THF, 2 mM NADH, 3 mM lipoic acid, 5 mM ATP, 0.25 mM PLP, 10 mM DTT, 5 mM MgCl_2_, 1 mg·mL^−1^ *Am*FchA-MtdA, and 400 μg·mL^−1^ GcvP-GcvH-GcvT-GcvL. The reaction temperature was maintained at 30 °C.

### 2.6. Synthesis of Glycine Using the Control Strains

Subsequently, the whole-cell reaction of RGP was verified. First, we used the background strain, *E. coli* BL21; the control strain, strain B; or strain C in a one-pot, whole-cell catalysis method to produce glycine from formic acid. Since PPK2 can use PolyP as a phosphate donor for the synthesis and regeneration of ATP [28], to ensure the smooth progress of the reaction, *Rs*PPK2 was added to promote ATP generation. We separately extracted the wet cells of strain B, strain C, and *E. coli* BL21/RSFDuet-1-*Rs*PPK2 to participate in the reaction. The reaction mixture (1 mL) contained 100 mM sodium phosphate buffer (pH 7.0); 10 mM formate; 50 mM NaHCO_3_; 50 mM NH_4_Cl; 2 mM THF; 2 mM NADH; 3 mM lipoic acid; 5 mM ATP; 0.25 mM PLP; 10 mM DTT; 5 mM MgCl_2_; 10 mM sodium tripolyphosphate (STPP); 0.5 g of wet cells of *E. coli* BL21, strain B, or strain C; and 0.25 g of wet cells of *E. coli* BL21/*Rs*PPK2. The reaction temperature was maintained at 30 °C.

### 2.7. Synthesis of Glycine Using Strain D

To further enhance the production of glycine, we overexpressed FtfL and then employed strain D (*Nh*FtfL-*Am*FchA-MtdA-GcvP-GcvH-GcvT-GcvL) for one-pot, whole-cell catalysis to generate glycine from formic acid. During this period, we conducted comparative experiments using three different reaction conditions. The first reaction condition was the original system, where the reaction mixture (1 mL) contained 100 mM sodium phosphate buffer (pH 7.0), 10 mM formate, 50 mM NaHCO_3_, 50 mM NH_4_Cl, 2 mM THF, 2 mM NADH, 3 mM lipoic acid, 5 mM ATP, 0.25 mM PLP, 10 mM DTT, 5 mM MgCl_2_, 10 mM STPP, 0.5 g of wet cells of strain D, and 0.25 g of wet cells of *E. coli* BL21/*Rs*PPK2. The second reaction condition replaced STPP with sodium hexametaphosphate (SHMP). The reaction mixture (1 mL) contained 100 mM sodium phosphate buffer (pH 7.0), 10 mM formate, 50 mM NaHCO_3_, 50 mM NH_4_Cl, 2 mM THF, 2 mM NADH, 3 mM lipoic acid, 5 mM ATP, 0.25 mM PLP, 10 mM DTT, 5 mM MgCl_2_, 10 mM SHMP, 0.5 g of wet cells of strain D, and 0.25 g of wet cells of *E. coli* BL21/*Rs*PPK2. The reaction temperature was maintained at 30 °C.

### 2.8. Synthesis of Glycine Using Strain E

To further optimize the whole-cell reaction of RGP, we introduced RSFDuet-1-*Rs*PPK2 into strain D and then used strain E (*Nh*FtfL-*Am*FchA-MtdA-GcvP-GcvH-GcvT-GcvL-*Rs*PPK2) for the one-pot, whole-cell catalysis of formic acid to produce glycine. The reaction mixture (1 mL) contained 100 mM sodium phosphate buffer (pH 7.0), 10 mM formate, 50 mM NaHCO_3_, 50 mM NH_4_Cl, 2 mM THF, 2 mM NADH, 3 mM lipoic acid, 5 mM ATP, 0.25 mM PLP, 10 mM DTT, 5 mM MgCl_2_, 10 mM STPP, and 0.5 g of wet cells of strain E. The reaction temperature was maintained at 30 °C.

### 2.9. Direct Synthesis of Glycine from CO_2_ and NH_4_Cl

Whole-cell electrocatalytic synthesis of glycine from CO_2_ and NH_4_Cl was performed using strain D533S/E684I and strain E. The electro–whole-cell-coupled catalytic reaction was conducted in an H-type electrochemical cell. A graphite rod (diameter, 6 mm; length, 90 mm) served as the working electrode, while a platinum sheet (5 mm × 5 mm × 0.1 mm) functioned as the counter electrode (placed in the anode chamber). An Ag/AgCl reference electrode (3.5 M KCl; dimensions, 6 mm × 65 mm; potential, +198 mV vs. NHE) was used. A proton exchange membrane (PEM: Nafion 115; diameter, 30 mm; thickness, 0.125 mm) was positioned between the anode and cathode chambers. The three-electrode system was connected to an electrochemical workstation for data acquisition and analysis. The anode chamber was filled with 1 mM H_2_SO_4_ solution, providing protons that migrated across the PEM into the cathode chamber. The cathode chamber (containing both working and reference electrodes) was supplied with CO_2_ at a flow rate of 40 mL/min and contained the following components in 100 mM sodium phosphate buffer (pH 7.0): 20 mM NaHCO_3_, 50 mM NH_4_Cl, 0.5 mM THF, 2 mM NADH, 2 mM NADPH, 3 mM lipoic acid, 0.5 mM ATP, 10 mM STPP, 0.25 mM PLP, 10 mM DTT, 5 mM MgCl_2_, 0.5 g wet cell weight each of strain D533S/E684I and strain E, and 5 mM ethyl viologen. The reaction temperature was maintained at 30 °C with gentle stirring using a magnetic stirrer.

Cyclic voltammetry (CV) was employed to analyze the electro–whole-cell-coupled catalytic system. Samples were collected from the cathode chamber at 2 h intervals (2, 4, 6, 8, and 10 h). Glycine quantification was performed using the HPLC method described subsequently.

### 2.10. Detection Methods for Glycine

Precisely measure 1 mL of the test solution and transfer it into a 5 mL brown volumetric flask. Add 1.5 mL of 5% sodium bicarbonate aqueous solution and 0.6 mL of 1% 2,4-dinitrofluorobenzene (DNFB) acetonitrile solution. Shake well, place in a water bath at 60 °C, and react in the dark for 60 min. Then, take it out, dilute it with acetonitrile to the mark, shake well, centrifuge at 1000 rpm for 1 min, and filter through a 0.22 μm microporous membrane to obtain the sample. Chromatographic column: Thermo Scientific Hypersll^TM^ C18 (250 mm × 4.6 mm, 5 μm). Mobile phase: 0.05 mol·L^−1^ sodium acetate buffer salt (adjust the pH to 5.5 with hydrochloric acid)–acetonitrile (80:20). Injection volume: 10 μL. Flow rate: 1.0 mL·min^−1^. Column temperature: 35 °C. Detection wavelength: 360 nm [29].

### 2.11. Statistical Analysis

All experiments were performed in triplicate. Analysis of significant differences was conducted through Bonferroni’s multiple comparison test in GraphPad Prism 8.0 [1,2].

## 3. Results and Discussion

### 3.1. Gene Cloning and Expression Identification of Each Enzyme in RGP

**Figure 2:**
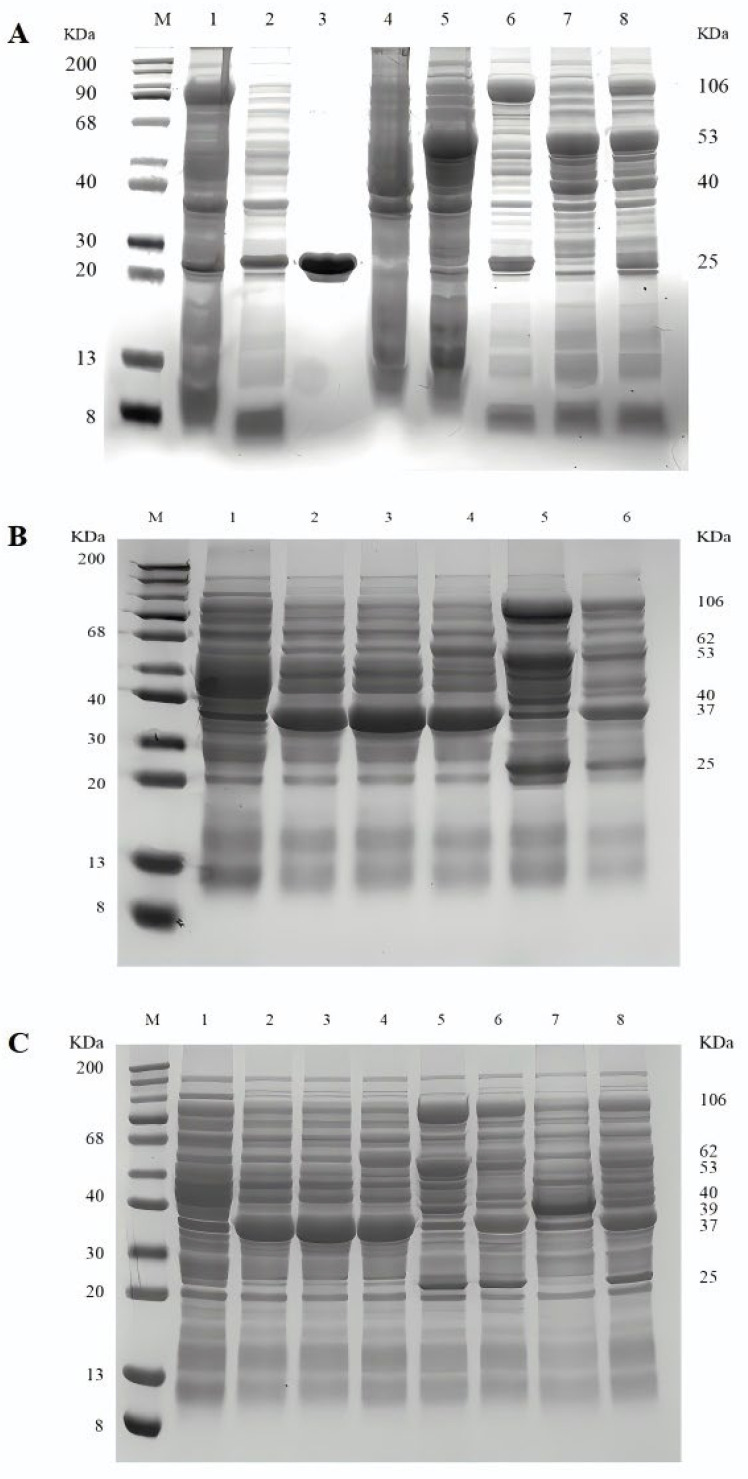
**SDS-PAGE analysis.** (**A**) **SDS-PAGE analysis of strain A.** M, 200 kDa Marker; 1, *E. coli* BL21/pACYCDuet-1-GcvP crude enzyme; 2, *E. coli* BL21/pACYCDuet-1-GcvH crude enzyme; 3, *E. coli* BL21/pACYCDuet-1-GcvH purified enzyme; 4, *E. coli* BL21/pETDuet-1-GcvT crude enzyme; 5, *E. coli* BL21/pETDuet-1-GcvL crude enzyme; 6, *E. coli* BL21/pACYCDuet-1-GcvP-GcvH crude enzyme; 7, *E. coli* BL21/pETDuet-1-GcvT-GcvL crude enzyme; and 8, *E. coli* BL21/pACYCDuet-1-GcvP-GcvH: pETDuet-1-GcvT-GcvL crude enzyme. (**B**) **SDS-PAGE analysis of strain D.** M, 200 kDa Marker; 1, *E. coli* BL21 blank control; 2, *E. coli* BL21/pCDFDuet-1-*Am*FchA-MtdA free enzyme; 3, *E. coli* BL21/pCDFDuet-1-*Nf*FchA-MtdA free enzyme; 4, *E. coli* BL21/pCDFDuet-1-*Nh*FtfL-*Am*FchA-MtdA free enzyme; 5, *E. coli* BL21/pACYCDuet-1-GcvP-GcvH: pETDuet-1-GcvT-GcvL free enzyme; and 6, *E. coli* BL21/pCDFDuet-1-*Nh*FtfL-*Am*FchA-MtdA: pACYCDuet-1-GcvP-GcvH: pETDuet-1-GcvT-GcvL free enzyme. (**C**) **SDS-PAGE analysis of strain E.** M, 200 kDa Marker; 1, *E. coli* BL21 blank control; 2, *E. coli* BL21/pCDFDuet-1-*Am*FchA-MtdA free enzyme; 3, *E. coli* BL21/pCDFDuet-1-*Nf*FchA-MtdA free enzyme; 4, *E. coli* BL21/pCDFDuet-1-*Nh*FtfL-*Am*FchA-MtdA free enzyme; 5, *E. coli* BL21/pACYCDuet-1-GcvP-GcvH: pETDuet-1-GcvT-GcvL free enzyme; 6, *E. coli* BL21/pCDFDuet-1-*Nh*FtfL-*Am*FchA-MtdA: pACYCDuet-1-GcvP-GcvH: pETDuet-1-GcvT-GcvL free enzyme; 7, *E. coli* BL21/RSFDuet-1-*Rs*PPK2 free enzyme; and 8, *E. coli* BL21/RSFDuet-1-*Rs*PPK2: pCDFDuet-1-*Nh*FtfL-*Am*FchA-MtdA: pACYCDuet-1-GcvP-GcvH: pETDuet-1-GcvT-GcvL free enzyme.

First, the GcvP and GcvH genes were cloned into the pACYCDuet-1 vector, respectively, and the GcvT and GcvL genes were cloned into the pETDuet-1 vector, respectively, for heterologous expression in *E. coli*. Then, the GcvP and GcvH genes were cloned into the pACYCDuet-1 vector simultaneously, and the GcvT and GcvL genes were cloned into the pETDuet-1 vector simultaneously for heterologous expression in *E. coli*. Finally, the dual-expression genes pACYCDuet-1-GcvP-GcvH and pETDuet-1-GcvT-GcvL were co-transformed into a competent cell to achieve heterologous expression. As shown in Figure 2A, GcvP, GcvH, GcvT, and GcvL achieved soluble expression with apparent molecular weights of approximately 106, 25, 40, and 53 kDa, respectively.

The two composite genes, *Am*FchA-MtdA and *Nf*FchA-MtdA, were respectively digested and ligated into the pCDFDuet-1 vector, as shown in Figure S3. Then, *Nh*FtfL was digested and ligated onto the pCDFDuet-1-*Am*FchA-MtdA vector for heterologous expression in *E. coli*, as shown in Figure S4. Subsequently, it was introduced into the competent cells of strain A to achieve heterologous expression. As shown in Figure 2B, soluble expressions of *Nh*FtfL, *Am*FchA-MtdA, *Nf*FchA-MtdA, GcvP, GcvH, GcvT, and GcvL were obtained with apparent molecular weights of approximately 62, 37, 37, 106, 25, 40, and 53 kDa, respectively.

The *Rs*PPK2 was digested and ligated onto the RSFDuet-1 vector for heterologous expression in *E. coli*. Then, it was introduced into the competent cells of strain D to achieve heterologous expression. As shown in Figure 2C, soluble expressions of *Rs*PPK2, *Nh*FtfL, *Am*FchA-MtdA, *Nf*FchA-MtdA, GcvP, GcvH, GcvT, and GcvL were obtained with apparent molecular weights of approximately 39, 37, 37, 106, 25, 40, and 53 kDa, respectively. Thus, it can be concluded that all the enzymes in the RGP were successfully constructed and expressed, as shown in Figures S5–S7.

### 3.2. Enzyme Activity Assay and Characterization of Enzymatic Properties

**Figure 3:**
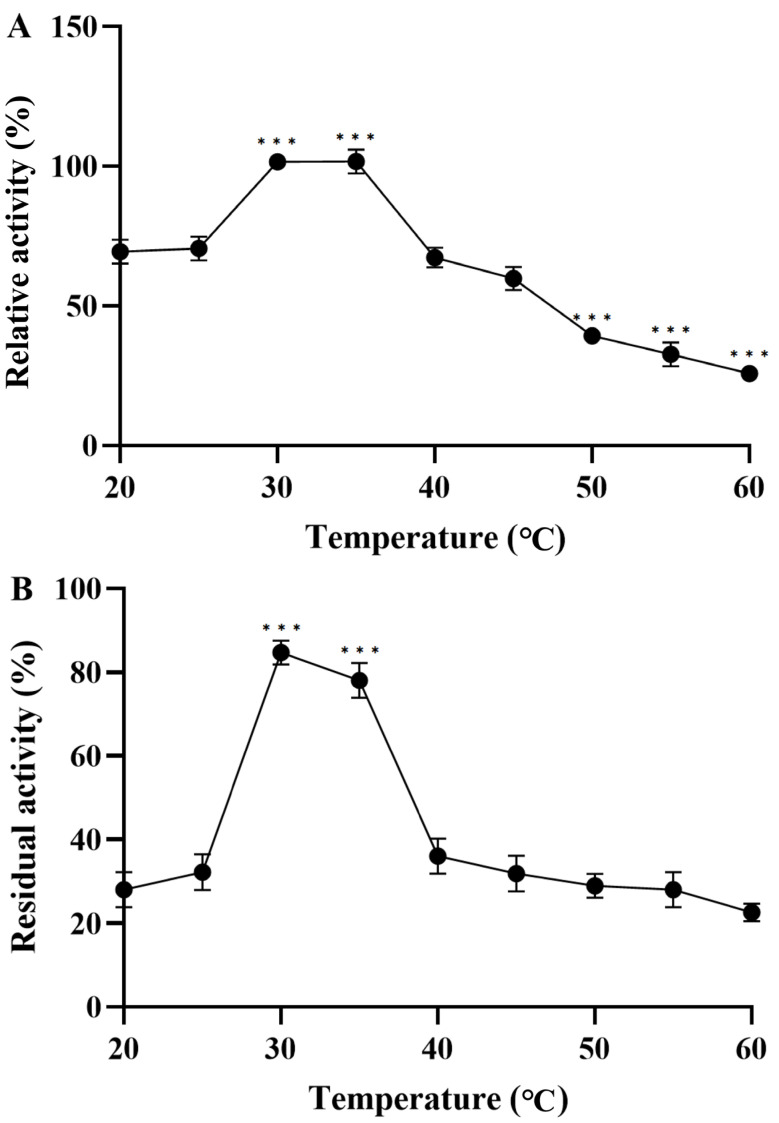
**Enzymatic properties of GcvP.** (**A**) **Optimal temperature:** The optimal reaction temperature of GcvP was determined under the aforementioned standard assay conditions, with a temperature range of 20–60 °C. (**B**) **Thermostability:** GcvP was incubated at temperatures ranging from 20 to 60 °C for 1 h. The GcvP enzymes’ residual reductive activities were measured at their respective optimal temperature. Three asterisks (***) denote significance at *p* < 0.001. Analysis of significant differences was conducted through the Bonferroni multiple comparison test.

The enzymatic activity of GCS for catalyzing glycine cleavage (single-protein assay) is 3.9 mU/mL, the enzymatic activity of GCS for catalyzing glycine cleavage (assayed using strain A) is 6.5 mU/mL, and the enzymatic activity of GCS for catalyzing glycine synthesis (assayed using strain A: *E. coli* BL21/pACYCDuet-1-GcvP-GcvH: pETDuet-1-GcvT-GcvL) is 1.7 mU/mL. The enzymatic activity of GcvP for catalyzing glycine cleavage is 0.6 U/mL, and its specific activity is 0.52 U/mg. Meanwhile, the enzymatic activity of GcvP for catalyzing glycine synthesis (at 30 °C) is 1.3 mU/mL, and its specific activity is 0.13 mU/mg. Although all reactions from formate to glycine are reversible, its reductase activity is greater than its oxidase activity, which also confirms that its overall thermodynamics favors the reduction direction (Δr*G*^’m^ < 0 KJmol^−1^) [30]. Subsequently, the optimal temperature and temperature stability of GcvP reduction were determined. As shown in Figure 3A, the optimal temperature for GcvP reduction is 30 °C, and its enzymatic activity is 1.3 mU/mL. GcvP was incubated at temperatures ranging from 20 to 60 °C for 1 h to test its temperature stability. As shown in Figure 3B, after incubating GcvP at 30 °C for 1 h, its residual activity can remain at nearly 82%. Evidently, conducting the reaction at 30 °C facilitates the activation of GcvP.

### 3.3. Synthesis of Glycine by GCS

**Figure 4:**
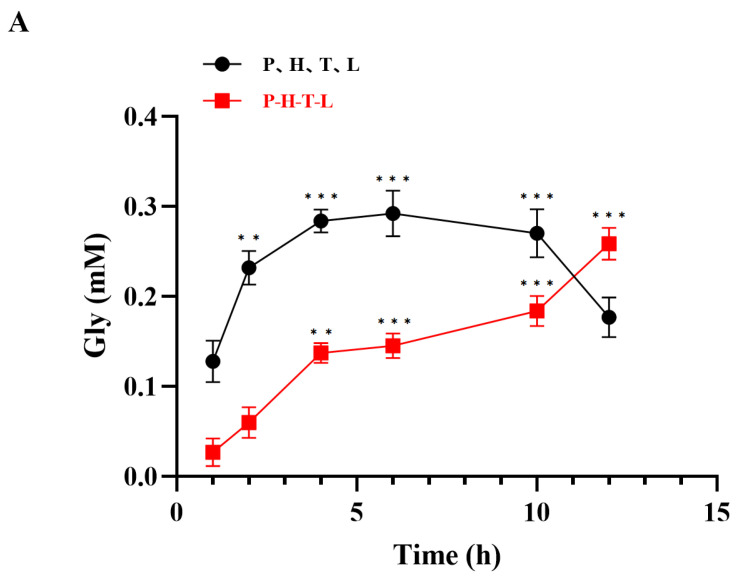

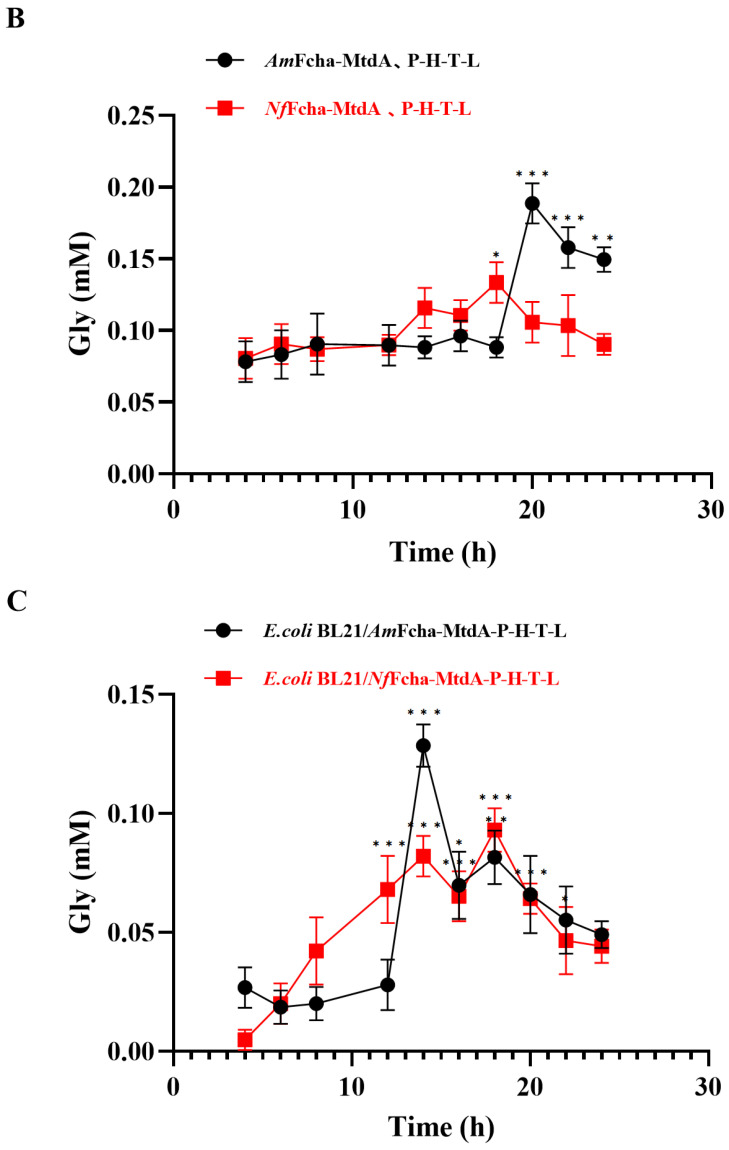

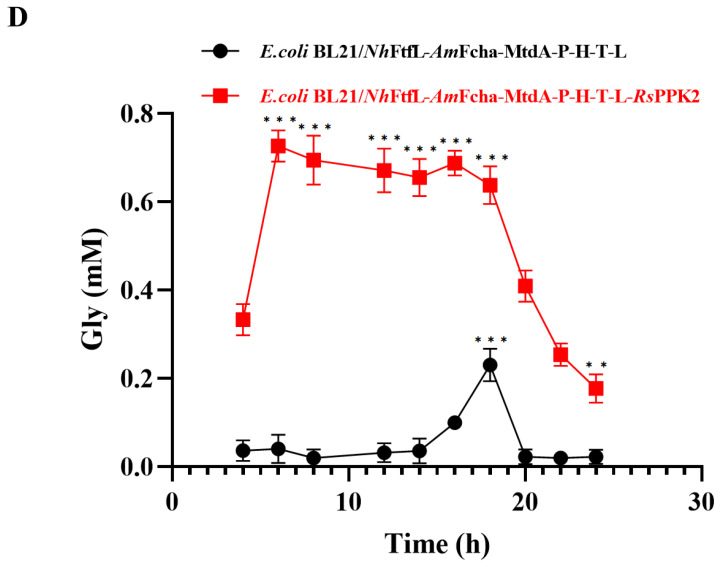
**Validation of one-pot glycine synthesis.** (**A**) Quantification of glycine at 1, 2, 4, 6, 10, and 12 h using individual free enzymes of the glycine cleavage system (GCS: GcvP, GcvH, GcvT, and GcvL) and free enzymes of strain A (GcvP-GcvH-GcvT-GcvL). (**B**) In vitro multi-enzyme cascade reaction using two different sources of composite free enzymes (*Am*FchA-MtdA/*Nf*FchA-MtdA) and free enzymes of strain A (GcvP-GcvH-GcvT-GcvL) to catalyze glycine synthesis from formate as substrate at 4, 6, 8, 10, 12, 14, 18, 20, 22, and 24 h. (**C**) In vivo quantification of glycine from formate as substrate at 4, 6, 8, 10, 12, 14, 18, 22, and 24 h using the engineered *E. coli* BL21, strain B (*Am*FchA-MtdA-GcvP-GcvH-GcvT-GcvL), or strain C (*Nf*FchA-MtdA-GcvP-GcvH-GcvT-GcvL). No glycine was detected in the reaction system catalyzed by the *E. coli* BL21 strain. (**D**) Comparative analysis of glycine quantification from formate as substrate at 4, 6, 8, 10, 12, 14, 18, 22, and 24 h using strain D (*E. coli* BL21/pCDFDuet-1-*Nh*FtfL-*Am*FchA-MtdA: pACYCDuet-1-GcvP-GcvH: pETDuet-1-GcvT-GcvL) and strain E (*E. coli* BL21/pCDFDuet-1-*Nh*FtfL-*Am*FchA-MtdA: pACYCDuet-1-GcvP-GcvH: pETDuet-1-GcvT-GcvL: RSFDuet-1-*Rs*PPK2). An asterisk (*) denotes statistical significance at *p* < 0.05, two asterisks (**) denote significance at *p* < 0.01, three asterisks (***) denote significance at *p* < 0.001. Analysis of significant differences was conducted through the Bonferroni multiple comparison test.

We employed the four key enzymes of the GCS (GcvP, GcvH, GcvT, and GcvL) and the free enzymes of strain A (GcvP-GcvH-GcvT-GcvL) to generate glycine from 5,10-methylenetetrahydrofolate. As shown in Figure 4A, when the four key enzymes of the GCS were used for the reaction, glycine production increased with reaction time within the first 6 h but gradually declined thereafter. In contrast, when the free enzymes of strain A were employed, glycine production exhibited a steady upward trend throughout the reaction process. Xu et al. demonstrated that the glycine carboxylation reaction catalyzed by the P protein is the rate-limiting step in the overall GCS reaction. Increasing the proportion of Hox in the GCS protein not only significantly enhances the reaction rate in the glycine synthesis direction but also improves the affinity of GCS for the substrates NH_4_^+^ and CO_2_ [31]. Since strain A cannot regulate the concentration of GcvH, whereas the four key enzymes of the GCS reaction initially exhibit overexpression of GcvH, the flux of the RGP pathway in *E. coli* is increased. Consequently, before 11 h of reaction, glycine production via the GCS exceeded that of strain A, with a maximum detected glycine concentration of 0.27 mM. However, using strain A not only optimizes the complex multi-step reaction but also yields more stable results. By 12 h of reaction, glycine production reached 0.27 mM as well. Therefore, we recommend the use of strain A for stable production.

### 3.4. Synthesis of Glycine Using Free Enzymes

Figure 4B illustrates the multi-enzyme cascade reaction using two distinct enzymes, *Am*FchA-MtdA and *Nf*FchA-MtdA, with strain A (GcvP-GcvH-GcvT-GcvL) to catalyze glycine synthesis from formate as the substrate. As shown in the figure, the glycine concentration produced by *Am*FchA-MtdA reached 0.18 mM, demonstrating higher catalytic efficiency compared to *Nf*FchA-MtdA. After 20 h of reaction, the glycine concentration gradually declined, likely due to insufficient ATP supplementation. As the reaction progressed, the remaining ATP was unable to sustain the subsequent reactions effectively.

### 3.5. Glycine Synthesis Using Whole-Cell Systems

When the parental *E. coli* BL21 strain was used in the reaction, the glycine yield was undetectable by high-performance liquid chromatography (HPLC), and it is therefore not shown in the figure. Figure 4C compared the glycine production yields from formate using one-pot, whole-cell catalysis by control strain B or strain C (*Am*FchA-MtdA-GcvP-GcvH-GcvT-GcvL or *Nf*FchA-MtdA-GcvP-GcvH-GcvT-GcvL). The results demonstrate that the strain B system achieved a maximum yield of 0.14 mM at 14 h, whereas the strain C system reached only 0.099 mM, further confirming the superior catalytic efficiency of *Am*FchA-MtdA over *Nf*FchA-MtdA. Furthermore, we employed strain D (*Nh*FtfL-*Am*FchA-MtdA-GcvP-GcvH-GcvT-GcvL) for one-pot, whole-cell catalytic synthesis of glycine from formate and compared two distinct reaction conditions. Our investigation revealed that the addition of sodium tripolyphosphate to the reaction system significantly enhanced the efficiency of the RGP. Consequently, sodium tripolyphosphate was consistently utilized as the substrate for the extracellular ATP regeneration system in all subsequent experiments. Figure 4D compared glycine production between strain E (*Nh*FtfL-*Am*FchA-MtdA-GcvP-GcvH-GcvT-GcvL-*Rs*PPK2) and strain D (*Nh*FtfL-*Am*FchA-MtdA-GcvP-GcvH-GcvT-GcvL). Glycine production reached 0.27 mM when using strain D as the whole-cell biocatalyst. Strain E demonstrated superior performance, achieving a maximum yield of 0.75 mM within 6 h, and its space–time productivity reached 0.125 mmol/L/h. Figures S8 and S9 show the HPLC chromatograms of glycine products from selected reactions. This improvement likely stems from intracellular *Rs*PPK2 expression, eliminating the need for transmembrane ATP transport and thereby enhancing reaction kinetics. During this initial phase, all system components, including substrates, cofactors, and enzymes, were in their optimal states. Favorable cell membrane permeability facilitated rapid reaction initiation and efficient glycine synthesis. The observed decline in yield between 6 h and 17 h is likely attributable to NADH depletion, which subsequently activated endogenous cellular metabolism. The cells utilized formate or trace carbon sources present in the system to slowly regenerate NADH via intrinsic dehydrogenases. By 17 h, NADH concentration reached a critical threshold, reactivating the entire RGP. Following 17 h, glycine production declined again due to secondary NADH depletion, potential product inhibition, and gradual loss of cellular viability. In summary, strain E demonstrated superior performance in one-pot, whole-cell catalytic glycine synthesis when using formate as the substrate.

### 3.6. Whole-Cell Electrocatalytic Synthesis of Glycine

**Figure 5:**
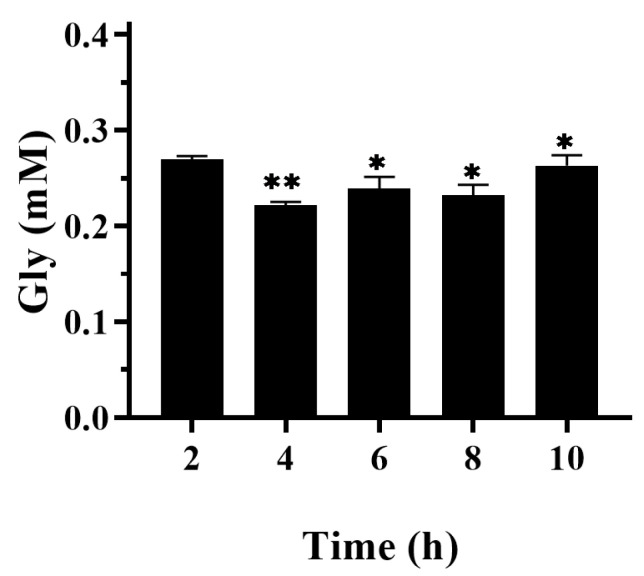
**Whole-cell electrocatalytic synthesis of glycine from CO_2_ and NH_4_Cl.** The reaction system contained CO_2_ and NH_3_ as substrates; wet cell biomass of strain D533S/E684I and strain E (*E. coli* BL21/pCDFDuet-1-*Nh*FtfL-*Am*FchA-MtdA: pACYCDuet-1-GcvP-GcvH: pETDuet-1-GcvT-GcvL: RSFDuet-1-*Rs*PPK2) as catalysts; and 10 mM phosphate buffer (pH 7.0), 20 mM NaHCO_3_, 50 mM NH_4_Cl, 0.5 mM THF, 2 mM NADH, 2 mM NADPH, 3 mM lipoic acid, 0.5 mM ATP, 10 mM STPP, 0.25 mM PLP, 10 mM DTT, 5 mM MgCl_2_, and 5 mM ethyl viologen. The temperature was maintained at 30 °C throughout the experiment. Samples were collected at 2, 4, 6, 8, and 10 h, respectively. An asterisk (*) denotes statistical significance at *p* < 0.05, two asterisks (**) denote significance at *p* < 0.01. Analysis of significant differences was conducted through the Bonferroni multiple comparison test.

Whole-cell electrocatalytic synthesis of glycine directly from CO_2_ and NH_4_Cl was performed using wet cells of strain D533S/E684I and strain E as biocatalysts. As shown in Figure 5, the glycine concentration reached a maximum of 0.27 mM at 2 h, and its space–time productivity reached 0.135 mmol/L/h. The subsequent instability in production may result from the dynamic interplay between electrochemical toxicity and cellular self-repair mechanisms. The CO_2_ conversion rate by strain D533S/E684I is limited to 4.1 mmol/L/h [26], resulting in insufficient formate production to adequately supply the subsequent RGP. This bottleneck directly constrains the overall glycine yield from CO_2_ and NH_4_Cl. CV is an electrochemical technique that records current response while controlling electrode potential at a constant scan rate. This method can be used to detect redox signals, verify the feasibility of redox reactions, and extract information about electron transfer processes at the electrode interface. In this study, CV further confirmed the occurrence of reduction reactions. Furthermore, CO_2_ dissolution involves the formation of multiple species whose distribution is influenced by solution pH and other physicochemical properties. Thus, electrons supplied from the anode may contribute to maintaining the thermodynamic equilibrium between different CO_2_ species and glycine. Figure S10 displays the CV obtained at a scan rate of 50 mV/s. The results demonstrate that the maximum reduction current reaches 3.18 mA, confirming the effective generation of reducing power in our electrochemical system.

This study successfully established a whole-cell electrocatalytic system for carbon and nitrogen co-assimilation, achieving, for the first time, de novo biosynthesis of glycine directly from CO_2_ and NH_4_Cl. Although the current titer (0.27 mM) requires further improvement, this system demonstrates the feasibility of converting greenhouse gases directly into high-value nitrogen-containing chemicals. It is particularly noteworthy that CO_2_ and NH_4_Cl serve as sustainable carbon and nitrogen sources [32], and their efficient conversion holds significant potential for enhancing atom economy and reducing waste emissions. Wu et al. [25] developed an in vitro multi-enzyme cascade system based on the RGP, enabling enzymatic electrocatalytic synthesis of glycine using CO_2_ and NH_3_ as the sole carbon and nitrogen sources. Under non-optimized conditions, their system produced approximately 0.3 mM glycine from 10 mM formate. In contrast, our reconstituted in vivo RGP achieved a glycine titer of 0.75 mM from the same substrate concentration—approximately 2.5-fold higher than the in vitro system. This disparity underscores the inherent advantages of whole-cell systems in cofactor regeneration, metabolite channeling, and operational stability, particularly in maintaining efficient multi-enzyme synergistic catalysis. However, the relatively low yield from direct CO_2_ and NH_4_Cl conversion is primarily limited by the catalytic efficiency of FDH, which restricts substrate supply. To address this, we propose employing nanomaterial-based immobilization techniques to enhance enzyme stability and reusability at the electrode interface. Notably, a recently developed chemoenzymatic system operating independently of ATP and NAD(P)H engineered the GCS by replacing NAD(P)H-dependent L-protein with dithiothreitol (DTT) as a chemical reductant and modifying the H-protein to enable lipoamide arm release. This system achieved high-yield glycine production (13.2 mM, 1.0 g/L) from captured atmospheric CO_2_ and methanol [5]. Inspired by this, we aim to explore alternative reducing power supply mechanisms independent of traditional cofactors and, most importantly, enhance pathway flux through directed evolution or rational design of rate-limiting enzymes (e.g., GcvP) in the RGP. Of particular significance, researchers have replaced the native Calvin cycle in Cupriavidus necator with a synthetic RGP, resulting in a 17% higher biomass yield on formate and CO_2_ compared to the wild-type strain exceeding that of any natural formatotroph relying on the Calvin cycle [33]. This study provides the first experimental evidence that a fully artificial synthetic metabolic pathway can outperform a natural pathway refined by billions of years of evolution in supporting microbial growth. Higher yield implies reduced C1 feedstock requirements for producing equivalent amounts of biomass (or derived chemicals), directly lowering production costs and enhancing the economic viability of sustainable electro-microbial production using formate and CO_2_. Moving forward, we plan to implement stable genomic integration of key pathway components to achieve higher conversion efficiency.

## 4. Conclusions

This study achieved systematic reconstruction of the RGP by constructing an engineered *E. coli* system (strain E) harboring four plasmids for coordinated expression of GcvP-GcvH, GcvT-GcvL, *Nh*FtfL-*Am*FchA-MtdA, and *Rs*PPK2. Enzymatic characterization demonstrated for the significant reductive preference of the rate-limiting enzyme GcvP in the GCS system (reductive activity > oxidative activity), with 30 °C identified as its optimal catalytic temperature, providing theoretical guidance for pathway optimization. In terms of catalytic performance, the developed one-pot, whole-cell catalytic system achieved a glycine concentration of 0.75 mM, representing a 2.5-fold improvement over the existing literature values. More significantly, we established the whole-cell electrocatalytic platform for direct conversion of CO_2_ and NH_3_ to glycine, obtaining 0.27 mM glycine and completing a full synthesis route from greenhouse gases to high-value nitrogen-containing chemicals. This research has developed self-sustaining metabolic networks through multi-plasmid coordination and energy module (*Rs*PPK2) integration, pioneering a new paradigm for whole-cell electrocatalytic glycine synthesis. The established methodology provides a transferable framework for complex metabolic pathway reconstruction. These advancements not only propel C1 biotransformation technology forward but also offer novel technical routes and theoretical support for green biomanufacturing and carbon resource valorization.

## Figures and Tables

**Table 1:** Recombinant strains used in the present study.

Strain Name	Plasmid	Function
A	*E. coli* BL21/pACYCDuet-1-GcvP-GcvH: pETDuet-1-GcvT-GcvL	5,10-methylene-THF → glycine
B	*E. coli* BL21/pCDFDuet-1-*Am*FchA-MtdA: pACYCDuet-1-GcvP-GcvH: pETDuet-1-GcvT-GcvL	10-formyl-THF → glycine
C	*E. coli* BL21/pCDFDuet-1-*Nf*FchA-MtdA: pACYCDuet-1-GcvP-GcvH: pETDuet-1-GcvT-GcvL	10-formyl-THF → glycine
D	*E. coli* BL21/pCDFDuet-1-*Nh*FtfL-*Am*FchA-MtdA: pACYCDuet-1-GcvP-GcvH: pETDuet-1-GcvT-GcvL	formate → glycine
E	*E. coli* BL21/RSFDuet-1-*Rs*PPK2: pCDFDuet-1-*Nh*FtfL-*Am*FchA-MtdA: pACYCDuet-1-GcvP-GcvH: pETDuet-1-GcvT-GcvL	formate → glycine; ADP → ATP

**Table 2:** Genes used in the present study.

Genes	Source	Accession Number
GcvP	*Escherichia coli*	WP_112929453.1
GcvH	*Enterobacteriaceae*	WP_001295377.1
GcvT	*Escherichia coli*	WP_099356926.1
GcvL	*Escherichia coli*	WP_110826218.1
*Nh*FtfL	*Neomoorella humiferrea*	WP_338835031.1
*Am*FchA-MtdA	*Alkaliphilus metalliredigens*	WP_012063162.1
*Nf*FchA-MtdA	*Natronincola ferrireducens*	WP_090553537.1
*Rs*PPK2	*Rhodobacter sphaeroides*	UniProt No. Q3IZT9

## Data Availability

The original contributions presented in this study are included in the article/Supplementary Materials. Further inquiries can be directed to the corresponding authors.

## Supplementary Materials

The following supporting information can be downloaded at https://www.mdpi.com/article/10.3390/microorganisms14010236/s1, Figure S1: multiple sequence alignment of FchA-MtdA; Figure S2: Gene cloning strategy for enzymes in rGlyP; Figure S3: agarose gel electrophoresis of double digestion for pCDFDuet-1 and FchA-MtdA; Figure S4: Agarose gel electrophoresis of double digestion for pCDFDuet-1-*Am*FchA-MtdA and *Nh*FtfL; Figure S5: Agarose gel electrophoresis of PCR products from the strain A; Figure S6: Agarose gel electrophoresis of PCR products from the strain D; Figure S7: Agarose gel electrophoresis of PCR products from the strain E; Figure S8: HPLC chromatogram of glycine standard; Figure S9: HPLC chromatogram of glycine produced by the whole-cell rGlyP catalytic system of Strain E; Figure S10: Cyclic voltammetry (CV) graph; Table S1: Strains and plasmids used for this study; Table S2: Primers used in this study.
