# Supplementary material for: Biosynthesis of Glycine from One-Carbon Resources Using an Engineered Escherichia coli Whole-Cell Catalyst
Source: Microorganisms. 2026 Jan 20;14(1):236. doi: 10.3390/microorganisms14010236 (PMC12844395; doi:10.3390/microorganisms14010236)
Supplement: Supplementary file 1 [file microorganisms-14-00236-s001.zip › microorganisms-4097815-supplementary.pdf]

Supplementary Information

Biosynthesis of Glycine from One-Carbon Resources Using an Engineered Escherichia coli Whole-Cell Catalyst

Muran Fu [1], Hongling Shi [1,2], Xueyang Bai [1], Qian Gao [1], Fei Liu [1], Dandan Li [1], Yunchao Kan [1], Chuang Xue [2], Lunguang Yao [1,*] and Cunduo Tang [1,*]

1 Henan Provincial Engineering Laboratory of Insect Bio-Reactor, Henan International Joint Laboratory of Insect Biology and Henan Key Laboratory of Insect Biology, College of Life Science, Nanyang Normal University, 1638 Wolong Road, Nanyang 473061, China

2 School of Bioengineering, Dalian University of Technology, 2 Linggong Road, Dalian 116024, China

***Correspondence:** 20081063@nynu.edu.cn (L.Y.); tcd530@nynu.edu.cn (C.T.); Tel./Fax: +86-377-63513726 (C.T.)

Number of pages: 14
Number of Figures: 10
Number of Tables: 2

Supplementary Information

Figure S1. Multiple sequence alignment of FchA-MtdA.

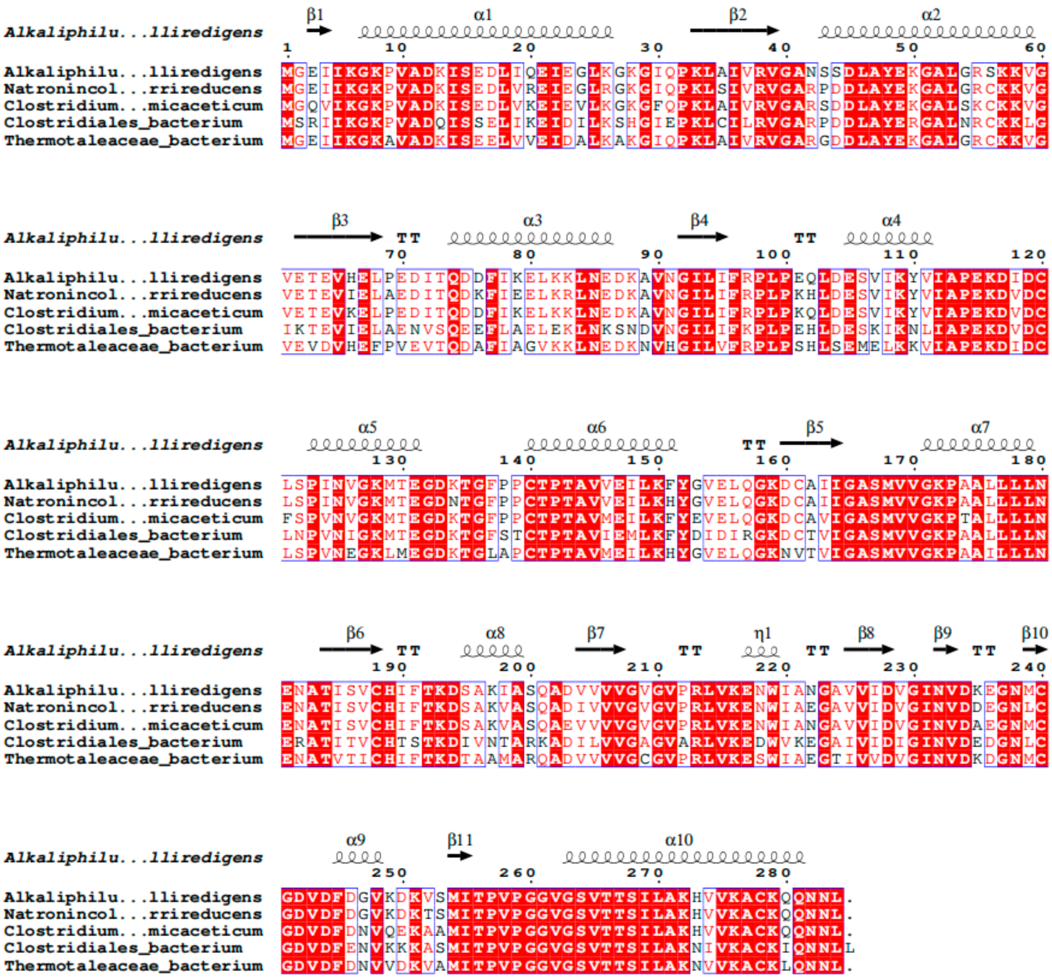

Figure S2. Gene cloning strategy for enzymes in rGlyP. Created with MedPeer (medpeer.cn). The strain A (GcvP-GcvH-GcvT-GcvL), harboring two dual-expression plasmids, was initially constructed using Gibson Assembly. Subsequently, restriction enzyme-based cloning was employed to generate the strain B (*Am*FchA-MtdA-GcvP-GcvH-GcvT-GcvL), strain C (*Nf*FchA-MtdA-GcvP-GcvH-GcvT-GcvL), and strain D (*Nh*FtfL-*Am*FchA-MtdA-GcvP-GcvH-GcvT-GcvL), each carrying three plasmids. Finally, the strain E (*Rs*PPK2-*Nh*FtfL-*Am*FchA-MtdA-GcvP-GcvH-GcvT-GcvL), containing four plasmids, was constructed via the same restriction-ligation method.

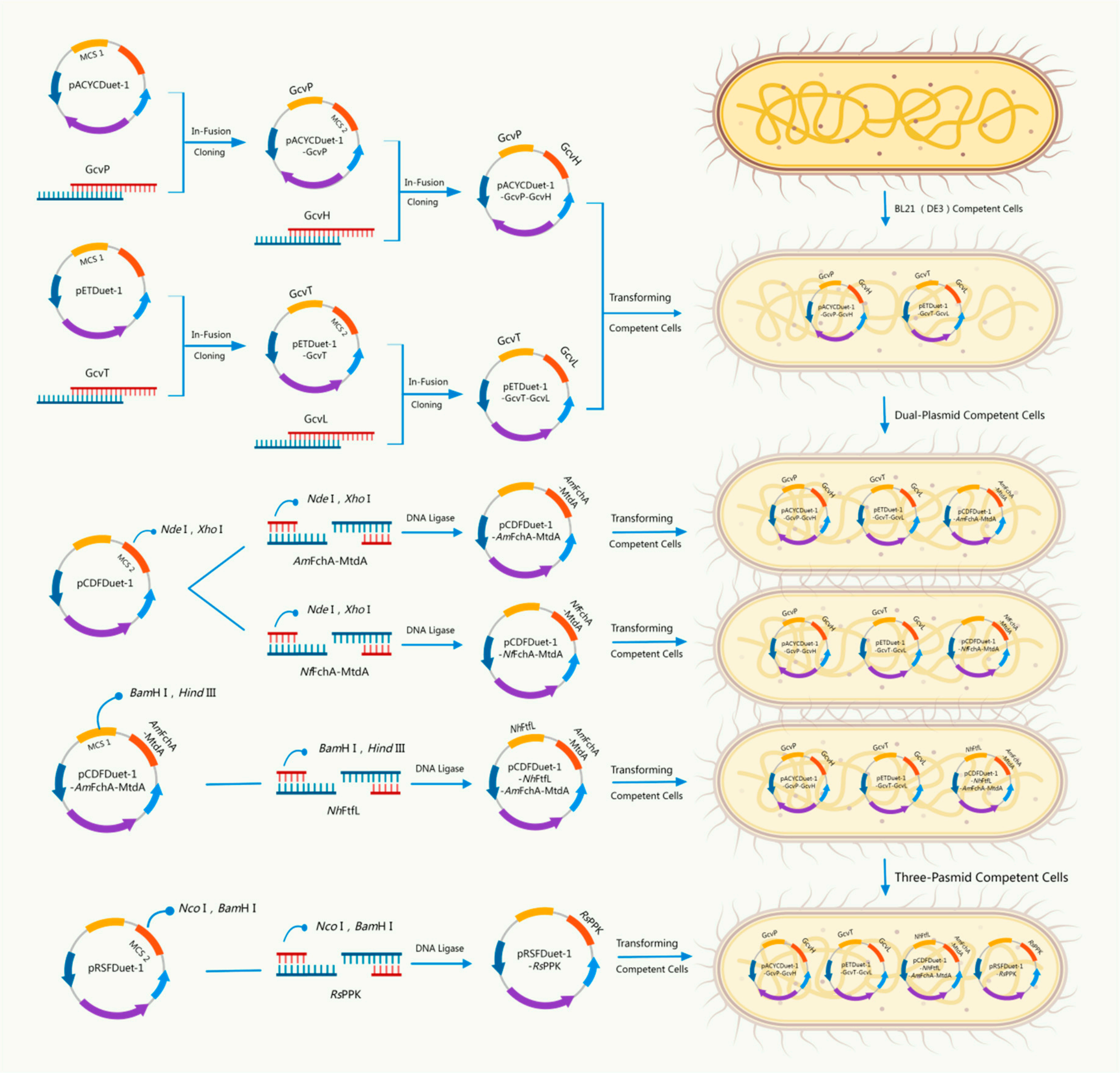

Figure S3. Agarose gel electrophoresis of double digestion for pCDFDuet-1 and FchA-MtdA. M: 5000 bp Marker; 1: pCDFDuet-1; 2: pCDFDuet-1; 3: pET-28a(+)-*Am*FchA-MtdA; 4: pET-28a(+)-*Nf*FchA-MtdA.

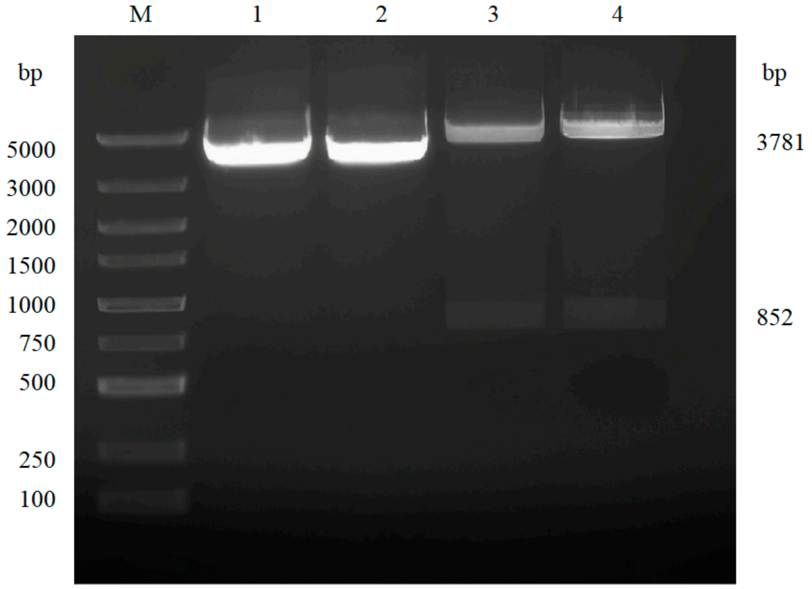

Figure S4. Agarose gel electrophoresis of double digestion for pCDFDuet-1-*Am*FchA-MtdA and *Nh*FtfL. M: 5000 bp Marker; 1: pCDFDuet-1-*Am*FchA-MtdA; 2: pET-28b(+)-*Nh*FtfL.

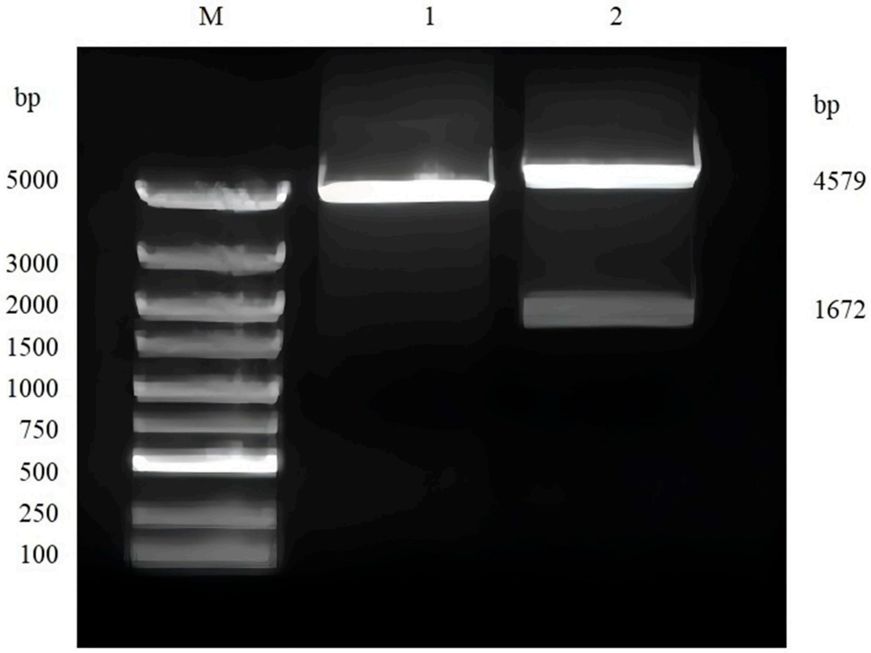

Figure S5. Agarose gel electrophoresis of PCR products from the strain A. M: 5000 bp Marker; 1: GcvP; 2: GcvH; 3: GcvT; 4: GcvL.

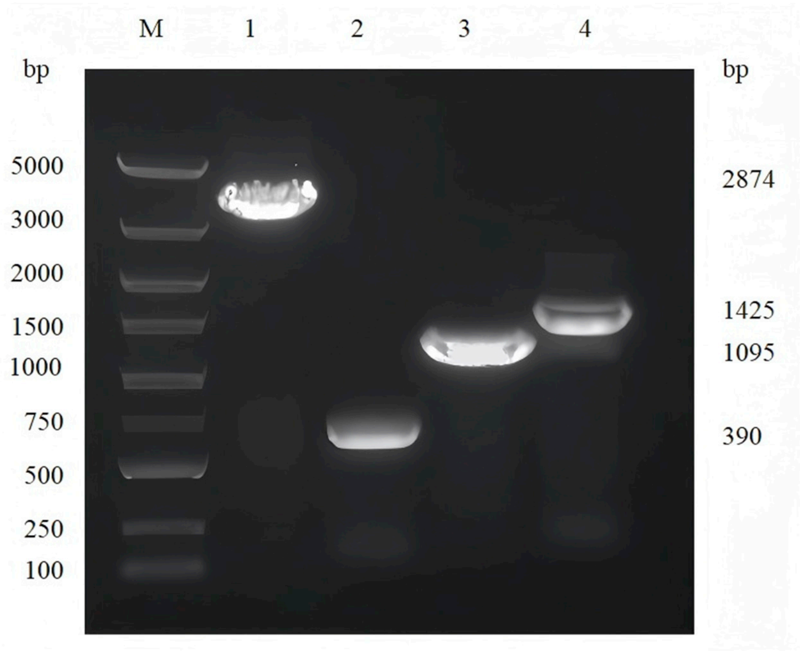

Figure S6. Agarose gel electrophoresis of PCR products from the strain D. M: 5000 bp Marker; 1: GcvP; 2: GcvH; 3: GcvT; 4: GcvL; 5: *Nh*FtfL; 6: *Am*FchA-MtdA.

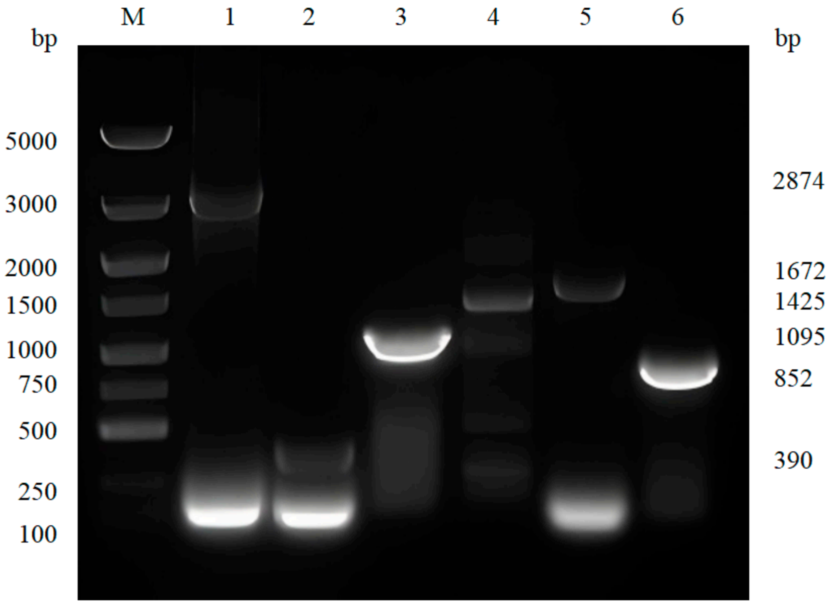

Figure S7. Agarose gel electrophoresis of PCR products from the strain E. M: 5000 bp Marker; 1: GcvP; 2: GcvH; 3: GcvT; 4: GcvL; 5: *Nh*FtfL; 6: *Am*FchA-MtdA; 7: *Rs*PPK2.

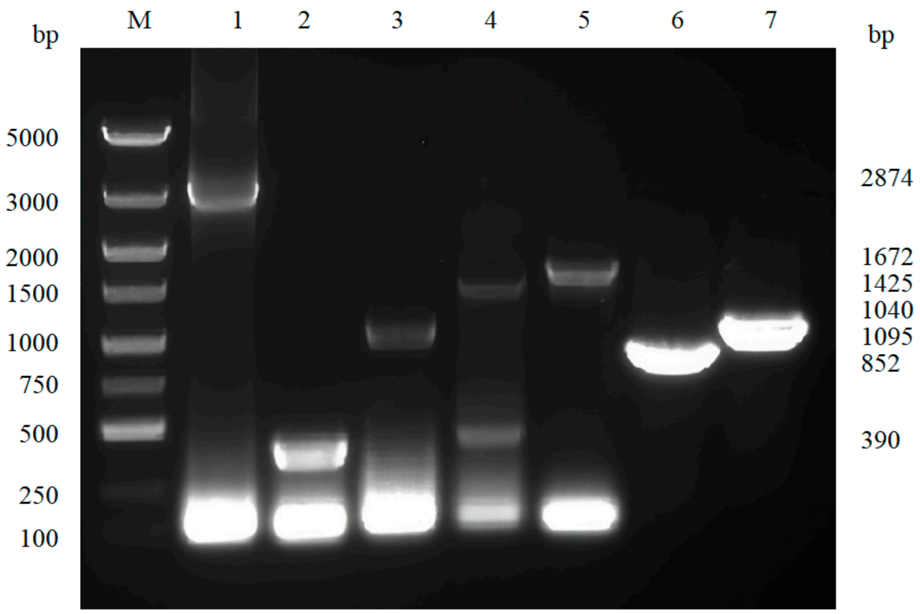

Figure S8. HPLC chromatogram of glycine standard. 1. 2,4-dinitrofluorobenzene (DNFB: 5.691 min); 2. Glycine (Gly: 6.422 min)

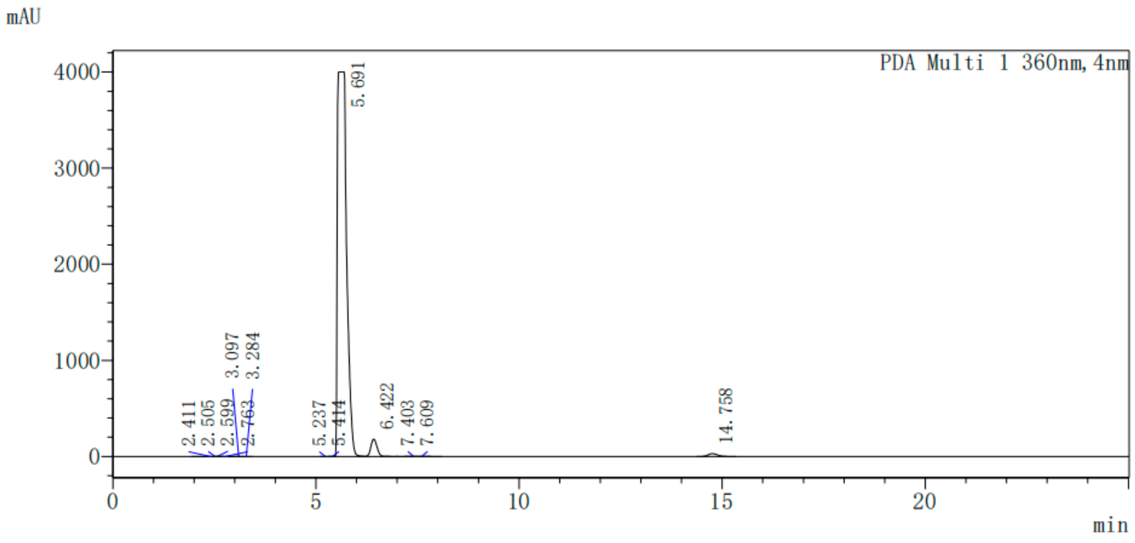

Figure S9. HPLC chromatogram of glycine produced by the whole-cell rGlyP catalytic system of Strain E. 1. 2,4-dinitrofluorobenzene (DNFB: 5.733 min); 2. Glycine (Gly: 6.474 min)

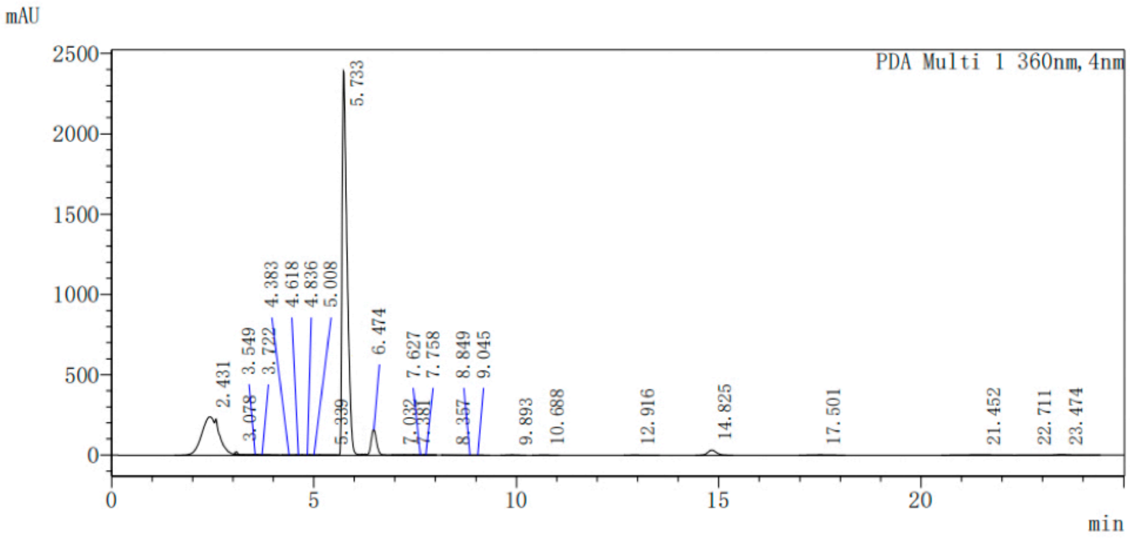

Figure S10. Cyclic voltammetry (CV) graph. At a scan rate of 50 mV/s, whole-cell electrocatalytic synthesis of glycine from CO_2 and NH_3 was conducted using wet cells of strain D533S/E684I and strain E as biocatalysts.

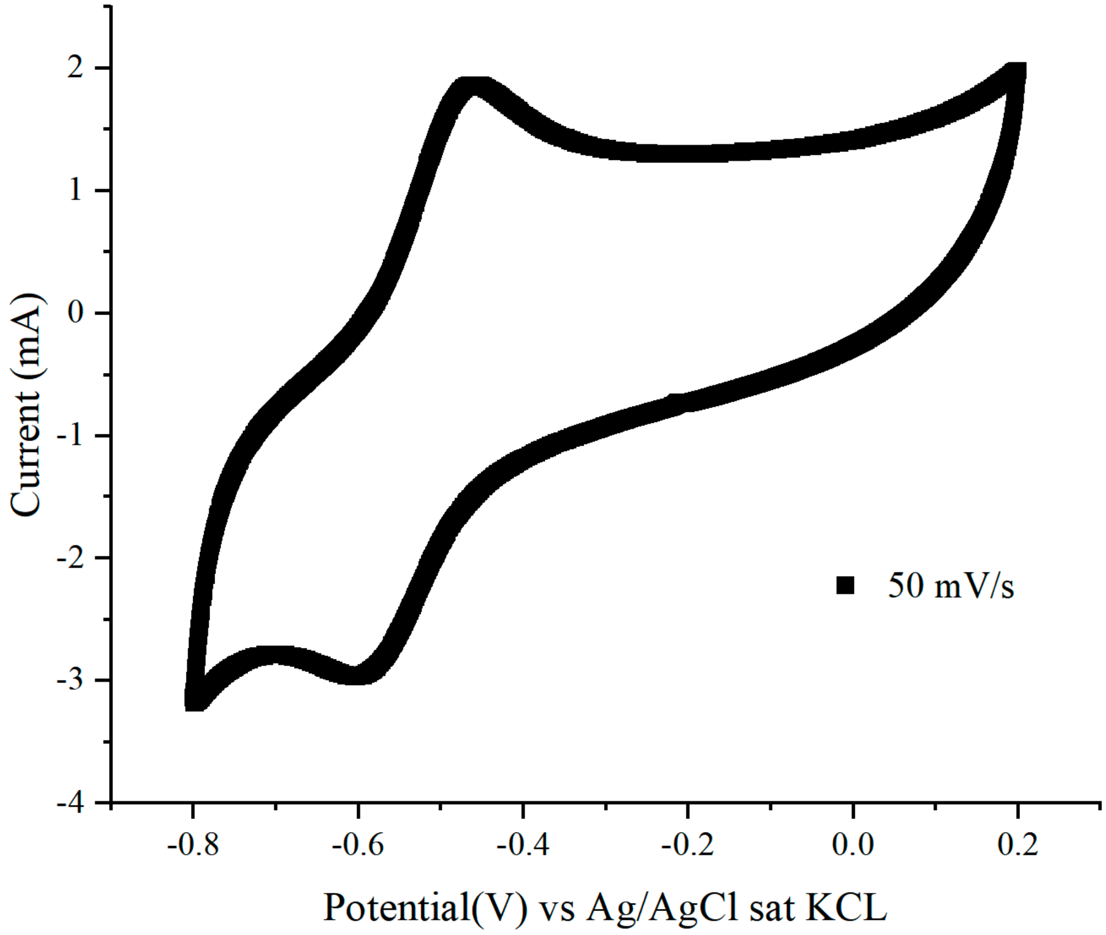

Table S1. Strains and plasmids used for this study

	Description	Reference
***E. coli* Strains**		
BL21(DE3)	Host for protein overexpression and purification	Sangon Ltd.
**Plasmids**		
pET-28a(+)	Plasmid for protein overexpression	Novagen
pET-28b(+)	Plasmid for protein overexpression	Novagen
pET-32a(+)	Plasmid for protein overexpression	Novagen
pACYCDuet-1	Plasmid for protein overexpression	Novagen
pETDuet-1	Plasmid for protein overexpression	Novagen
pCDFDuet -1	Plasmid for protein overexpression	Novagen
RSFDuet-1	Plasmid for protein overexpression	Novagen
pACYCDuet-1-GcvP	pACYCDuet-1 vector containing P-protein gene (NCBI No. WP_112929453.1)	This study
pACYCDuet-1-GcvH	pACYCDuet-1 vector containing H-protein gene (NCBI No. WP_001295377.1)	This study
pETDuet-1-GcvT	pETDuet-1 vector containing T-protein gene (NCBI No. WP_099356926.1)	This study
pETDuet-1-GcvL	pETDuet-1 vector containing L-protein gene (NCBI No. WP_110826218.1)	This study
pET-28b(+)-*Nh*FtfL	pET28b vector containing *Nh*FtfL gene (NCBI No. WP_338835031.1)	This study
pET-28a(+)-*Am*FchA-MtdA	pET28a vector containing *Am*FchA-MtdA gene (NCBI No. WP_012063162.1)	This study
pET-28a(+)-*Nf*FchA-MtdA	pET28a vector containing *Nf*FchA-MtdA gene (NCBI No. WP_090553537.1)	This study
pCDFDuet-1-*Am*FchA-MtdA	pCDFDuet-1 vector containing *Am*FchA-MtdA gene (NCBI No. WP_012063162.1)	This study
pCDFDuet-1-*Nf*FchA-MtdA	pCDFDuet-1 vector containing *Nf*FchA-MtdA gene (NCBI No. WP_090553537.1)	This study
pACYCDuet-1-*Rs*PPK2	pACYCDuet-1 vector containing *Rs*PPK2 gene (UniProt No. Q3IZT9)	This study
RSFDuet-1-*Rs*PPK2	RSFDuet-1 vector containing *Rs*PPK2 gene (UniProt No. Q3IZT9)	This study
pET-32a(+)-D533S/E684I	pET-32a vector containing D533S/E684I gene. D533S/E684I is the mutated gene of *Pb*FDH (NCBI No. WP_082435282.1).	This study

pACYCDuet-1-GcvP-GcvH	pACYCDuet-1 vector containing P-protein gene (NCBI No. WP_112929453.1) and H-protein gene (NCBI No. WP_001295377.1)	This study
pETDuet-1-GcvT-GcvL	pETDuet-1 vector containing T-protein gene (NCBI No. WP_099356926.1) and L-protein gene (NCBI No. WP_110826218.1)	This study
pCDFDuet-1-*Nh*FtfL-*Am*FchA-MtdA	pCDFDuet-1 vector containing *Nh*FtfL gene (NCBI No. WP_338835031.1) and *Am*FchA-MtdA gene (NCBI No. WP_012063162.1)	This study

Table S2. Primers used in this study

Primer	Sequence
T-protein-F	CATCACCATCATCACCACATGGCACAACAGACTCCTTTG
T-protein-R	ATCCTGGCTTCACGCGACGGCTTTACC
T-protein-vehicle-F	AGCCAGGATCCGAATTCGAGC
T-protein-vehicle-R	GTGGTGATGATGGTGATGGCTG
L-protein-F	CGCCAGCACATGGACTCGATGAGTACTGAAATCAAAACT
L-protein-R	TTAAGCTGCGCTAGTAGATTACTTCTTCTTCGCTTTCGG
L-protein-vehicle-F	TCTACTAGCGCAGCTTAATTAACCTAGGC
L-protein-vehicle-R	CGAGTCCATGTGCTGGCGTTC
P-protein-F	CATCACCATCATCACCACATGACACAGACGTTAAGCCAG
P-protein-R	CGAATTCGGATCCTGGCTTTACTGGTATTCGCTAATCGG
P-protein-vehicle-F	AGCCAGGATCCGAATTCGAGCT
P-protein-vehicle-R	GTGGTGATGATGGTGATGGCTG
H-protein-F	GCACATGGACTCGATGAGCAACGTACCAGCA
H-protein-R	TAAGCTGCGCTAGTAGATTACTCGTCTTCTAACAATGCT
H-protein-vehicle-F	TCTACTAGCGCAGCTTAATTAACCTAGGC
H-protein-vehicle-R	CGAGTCCATGTGCTGGCGTTCA
BamH I-H-F	CGCGGATCCATGAGCAACGTACCA
Xho I-H-R	CCGCTCGAGTTACTCGTCTTCTAACAA
*Nh*FtfL-F	GGATCCGATGCCCAGCGACATCGAA
*Nh*FtfL-R	AAGCTTTAGAATAGGCCGGTAATTACACC
*Am*FchA-MtdA-F	GGAATTCCATATGGGTGAAATTATCAAGGGCA
*Am*FchA-MtdA-R	CCGCTCGAGCAGATTATTCTGCTGTTTG
*Nf*FchA-MtdA-F	GGAATTCCATATGGGTGAAATTATCAAGGGTAAACCGG
*Nf*FchA-MtdA-R	CCGCTCGAGCAGATTATTCTGCTGTTTGC
